# Oral/oropharyngeal “selfies” in gay and bisexual men: a pilot study exploring oropharyngeal screening for HPV-related possible malignancies

**DOI:** 10.3389/fpubh.2023.1233274

**Published:** 2023-09-14

**Authors:** Michael W. Ross, Sarah L. Bennis, C. Mark Nichols, I. Niles Zoschke, J. Michael Wilkerson, B. R. Simon Rosser, Cyndee L. Stull, Alan G. Nyitray, Charlene Flash, Samir S. Khariwala

**Affiliations:** ^1^Department of Family Medicine and Community Health, Medical School, University of Minnesota, Minneapolis, MN, United States; ^2^Division of Epidemiology and Community Health, School of Public Health, University of Minnesota, Minneapolis, MN, United States; ^3^Avenue 360 Clinic, Houston, TX, United States; ^4^School of Public Health, University of Texas Health Science Center at Houston, Houston, TX, United States; ^5^School of Dentistry, University of Minnesota, Minneapolis, MN, United States; ^6^Department of Psychiatry and Behavioral Medicine, Medical College of Wisconsin, Milwaukee, WI, United States; ^7^Department of Otolaryngology, Head and Neck Surgery, Medical School, University of Minnesota, Minneapolis, MN, United States

**Keywords:** human papillomavirus (HPV), gay, oropharyngeal cancer, screening, online, telemedicine, oral sex

## Abstract

**Objectives:**

This study aims to determine the potential uptake and quality of oropharyngeal “selfies” taken by gay/bisexual men as a screening approach for HPV-associated oropharyngeal cancer.

**Methods:**

From 1,699 gay/bisexual men in the US, surveyed about knowledge and attitudes to HPV-associated oropharyngeal cancer, a random sample of 320 men were invited to take an oropharyngeal “selfie” by smartphone and send it to the study website: 113 (35.5%) did so. Images were rated for quality by three healthcare professional raters blinded to each other's rating, with an otolaryngologist as the gold standard. In the second wave, those whose images were rated as unacceptable were sent a short instructional video and asked to send another image. Of the 65 invited, 46 did so. An additional 15.2% sent acceptable images, and a total of 28.3% of the sample was acceptable.

**Results:**

A total of 1,121 men willing to participate in the future study who believed they could take a quality “oral selfie” were potentially eligible for this activity. A random sample of 320 participated: 153 participants started (47.8%) and 113 participants (35.3%) submitted an image. Responders were more likely to be younger, have higher knowledge scores on oropharyngeal HPV-related cancer, and have had HPV vaccination. There was high agreement between the three raters. Images of good/acceptable quality were 22.1%; oropharynx partially occluded images were 29.2%; oropharynx not visible images were 18.6%; images too dark were 21.2%; and images too small were 8.8%. From the second wave of requests with instructional videos, an additional 15.2% sent in quality images, with the remaining issues being partial occlusion of the tonsils by the tongue.

**Conclusion:**

One-third of the invited gay and bisexual men sent oropharyngeal selfie images to the study website and a total of 28.3% were of clinically acceptable quality. Following an instructional video on poorer-quality images, additional quality images were received. One barrier, i.e., partial occlusion of the oropharynx by the tongue remained. Quality oropharyngeal “selfies” are obtainable online.

## Introduction

Oropharyngeal squamous cell cancers (OPSCC) associated with human papillomavirus (HPV) 16 infection are increasing in incidence and exceeded the incidence of high-risk HPV infection of the cervix in 2020 (1). HPV-related oropharyngeal malignancies differ from classic oropharyngeal malignancies by having a significantly better prognosis and lower associations with tobacco and alcohol use (1, 2). However, Heck et al. reported that the strongest risk factor for HPV markers in OPSCC in men was sex with another man in the past 5 years (OR = 8.89, 95% CI: 2.14–36.8) (3); furthermore, oral sex is also implicated (4).

The very significantly increased survival rates of HPV-related OPSCC, compared with non-HPV-associated OPSCC, have highlighted the importance of screening and early diagnosis. In a large US study of the National Cancer Database, 2-year overall survival rates for HPV-positive (caused by HPV) cases vs. HPV-negative cases (not caused by HPV) were 93.1 vs. 77.8% (*p* < 0.001) with an adjusted hazard ratio of 0.44 (95% CI: 0.36–0.53; *p* < 0.001) (5, 6). New approaches for screening and early detection in populations at high risk of developing HPV-related OPSCC are warranted.

Oropharyngeal malignancies have the potential diagnostic advantage of being visible to the inspection of the oropharynx (depending on the exact sub-site) by healthcare professionals including physicians, dentists, dental hygienists, and nurses. The use of visual images to diagnose cancer has been successfully utilized in Australia, where self-screening using smartphone images of potentially malignant skin lesions (including malignant melanoma) occurs (7). It was previously demonstrated that the diagnostic and treatment accuracy of store-and-forward malignant melanoma images is close to that of face-to-face clinical consultation (8).

In Western Europe, there have been reports of oral cancer rates significantly declining in men, while oropharyngeal cancer significantly increases (9). *Oral* inspections (of the oral cavity including the floor of the mouth, buccal surfaces, palate, and front two-thirds of the tongue) provide an opportunity for dental screening for oral malignancies, although only a very small proportion of these *oral* lesions are HPV related. We specifically differentiate here between *oral* (mouth, cheek, gums, and front of tongue) and *oropharyngeal* (including tonsils, uvula, back of the throat, and back of the tongue) cancers, and focus on *oropharyngeal* cancers because of the high proportion that are caused by HPV (9).

The combination of tobacco use, alcohol use, and HPV prevalence, which are all known risk factors for oropharyngeal cancer, puts GBM men at the intersection of several heightened risk factors. Tobacco use and alcohol use also add to the elevated risk of oral cancers, and tobacco-related and alcohol-related disorders are significantly more common among gay and bisexual men (GBM) than heterosexual men in nationally representative samples in the US (10, 11). It is also a reason to consider that the approaches investigated here may also apply to *oral* cancers, although that is not the focus of this study.

Artificial intelligence (AI) approaches have established that oral images depicting potentially malignant lesions can be identified with sensitivity and specificity approaching that of experienced clinicians (12). Information technology approaches to potentially malignant oral lesions have shown promise. Welikala et al. (12) used convolutional neural networks (CNNs), which are designed for processing structured arrays of data, including images, for referral for clinical decision-making. They demonstrated precision levels of 84.8% for lesion detection, and 67.5% for identifying the need to make referrals for conventional diagnosis. More recently, Tanriver et al. (13) used deep networks to develop second-stage classification analyses for the classification of oral lesions into benign lesions, potentially malignant lesions, and carcinomas.

Visual inspections of *oropharyngeal* sub-sites (palatine tonsils, tongue base, soft palate/uvula, and posterior oropharyngeal wall), where HPV-associated OPSCC occurs, however, have never been reported on images. Telemedicine advances during COVID-19 have shown that oral and oropharyngeal images can be taken in a clinical setting by patients with good results and are acceptable to patients (14, 15).

However, recent previous research with practitioners (16) has indicated that clinical screening for OPSCC in the US is an “orphan” practice, with physicians infrequently screening GBM for OPSCC unless symptoms are reported, while dentists may limit their inspection to the oral cavity but not the oropharynx. This is despite practitioners with significant numbers of GBM patients being aware of the heightened risk of HPV-associated OPSCC in GBM.

In view of the reported epidemic increase in HPV-associated and sexually transmissible cancer in the oropharynx, particularly in gay and bisexual men (1, 3, 4), we investigated whether smartphone “oral selfies,” of sufficient quality for screening, could be taken by GBM following online instruction.

## Methods

This cross-sectional study aimed to recruit 1,700 GBM from two online dating portals (Scruff and Jack'd; Perry Street Software Inc., New York, NY) used by GBM. In February–March 2022, GBM with a profile on either portal was shown a single advertisement with an embedded link to the online survey. The recruitment criteria were (a) GBM aged ≥18 years, who self-reported to be (b) living in the USA, (c) having sex with a man in the past 5 years, and (d) self-identified as men. Trans men, non-binary individuals, and other individuals self-identifying as men could participate. Interested individuals were directed to a screening questionnaire (Qualtrics, Provo, UT) to determine eligibility. If eligible, they were directed to the consent process, after which they could access the main survey.

All active users meeting eligibility criteria, who logged in during the 5-day campaign period saw the advertisement. Recruitment continued until the institutional review board (IRB) approved the number of participants who had responded and provided full consent (participants could pause the survey and continue later). Of the 9,264 total clicks, 4,192 were unique clicks on Scruff and 5,072 were unique clicks on Jack'd. Among these, 4,464 people commenced the consent process, 1,836 people completed the consent process (19.86% of unique clicks), and 114 participants were removed during the deduplication process. After validation, deduplication, and internal consistency protocols, 1,722 consenting participants remained eligible, and 1,699 completed the first question of the main survey.

All users in the US and its territories who logged in would see the ad, which could be saved as an inbox message to check later. The link could only be accessed by Scruff/Jack'd users. Scruff had a reach of 185,257 with 417,296 total impressions, whereas Jack'd had a reach of 120,409 with 247,956 impressions. Individuals who completed the survey were recompensed $50 for their effort.

After the recruitment period, all surveys were reviewed to determine uniqueness using a modified validation and deduplication protocol (17). Study materials were reviewed and approved by the University of Minnesota IRB.

**Figure 1:**
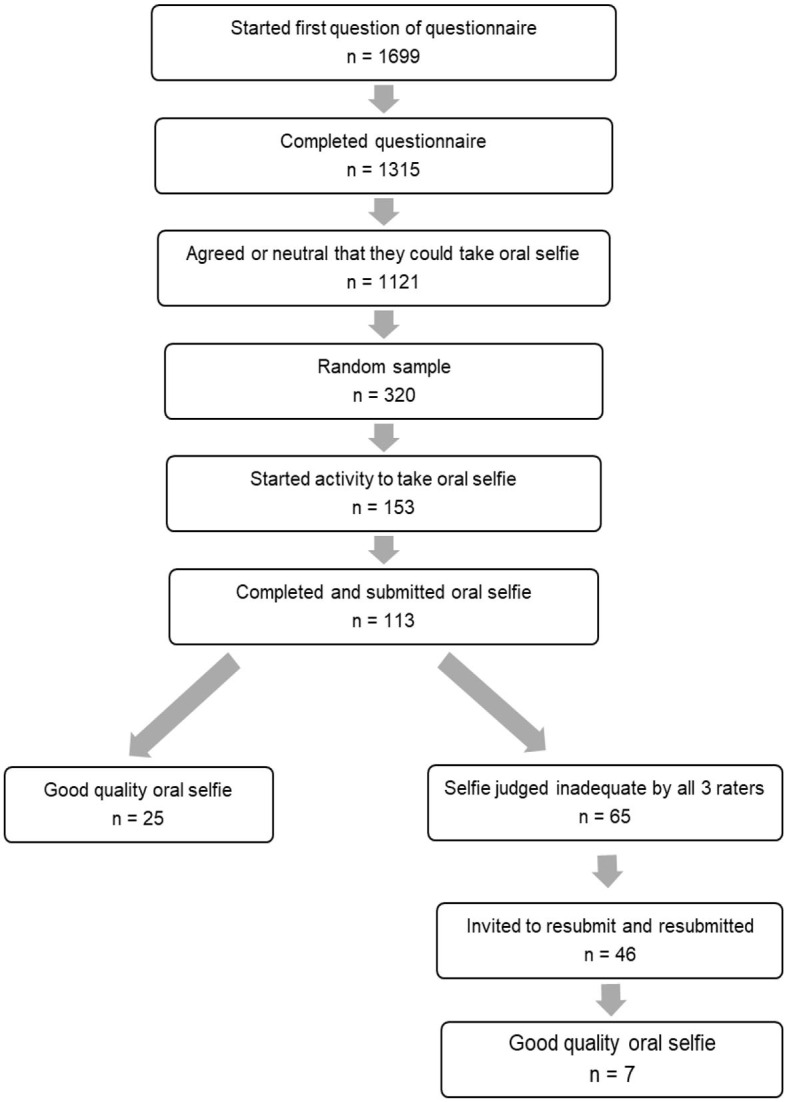
Flow of study design.

Participants from the survey who were willing to participate in future studies and “strongly agreed,” “somewhat agreed,” or “neither agreed nor disagreed” that they could take a quality “oral selfie” were potentially eligible (*n* = 1,121) for this activity. A random sample of 320 of these individuals was invited to participate. A total of 153 participants started the activity (47.8%) and 113 participants (35.3%) submitted an “oral selfie,” based on written instructions and an illustration of an ideal image, for rating. Figure 1 illustrates the sampling chain.

Participants who submitted one best “oral selfie” (multiple attempts possible) were compensated with a $30 Amazon gift card. Three clinicians blinded to each other's ratings (an otolaryngologist/head and neck surgeon, a dentist with a large GBM and HIV practice, and a medical researcher without clinical experience in the area) rated the photos. These photographs, in random order, were first rated for quality (good, acceptable, or unacceptable), and those considered not acceptable were additionally classified for one or more reasons: too dark, too small or unfocused, oropharynx partially occluded, or oropharynx not visible at all.

A second wave of those men whose images had not been rated by all of the raters as good or acceptable quality (to have no possibility that the selection of inadequate photos was a function of rating uncertainty, *n* = 65) was invited to watch a commercially produced 3-min video created for this purpose (https://vimeo.com/manage/videos/780429315) and take another oral selfie. The compensation was again $30. This study was approved by the University of Minnesota IRB. Differences between those who uploaded or did not upload a photo after being invited to participate in taking an “oral selfie” were computed using 2-tailed *t*-tests for interval data and χ^2^ tests for nominal or ordinal data.

## Results

Oropharyngeal oral selfies were received on the study website from 113 GBM. Comparing those who completed the request with those who did not (Table 1), those who completed the request were more likely to be younger, had higher HPV and oropharyngeal cancer knowledge scores, were more likely to have had HPV vaccination, and were more likely to have answered the question “Compared to other early cancer screenings, I believe that the benefits of checking for oropharyngeal cancer are higher.” There were no significant differences in race, ethnicity, sexual identity, or education.

**Table 1:** Differences between participants who completed an “oral selfie” and those who did not.

**Variable mean ±SD**	**Completed**	**Did not complete**	***p***
Age (years)	38.55 ± 11.44	42.62 ± 12.95 years	*t* = 2.80, df = 318, *p* = 0.006
HPV and oropharyngeal cancer knowledge scores^*^	5.20 ± 0.23	5.97 ± 0.29	*t* = 2.05, df = 317, *p* = 0.01
Had HPV vaccination (%)	45.53%	33.33%	χ^2^ = 4.61, df = 1, *p* = 0.03
Compared to other early cancer screenings, I believe that the benefits of checking for oropharyngeal cancer early are:^**^	3.97 ± 0.89	3.63 ± 0.90	χ^2^ = 12.88, df = 4, *p* = 0.012

* Possible range 0–12.

** 1 = Much lower; 2 = Somewhat lower; 3 = About the same; 4 = Somewhat higher; 5 = Much higher.

**Figure 2:**
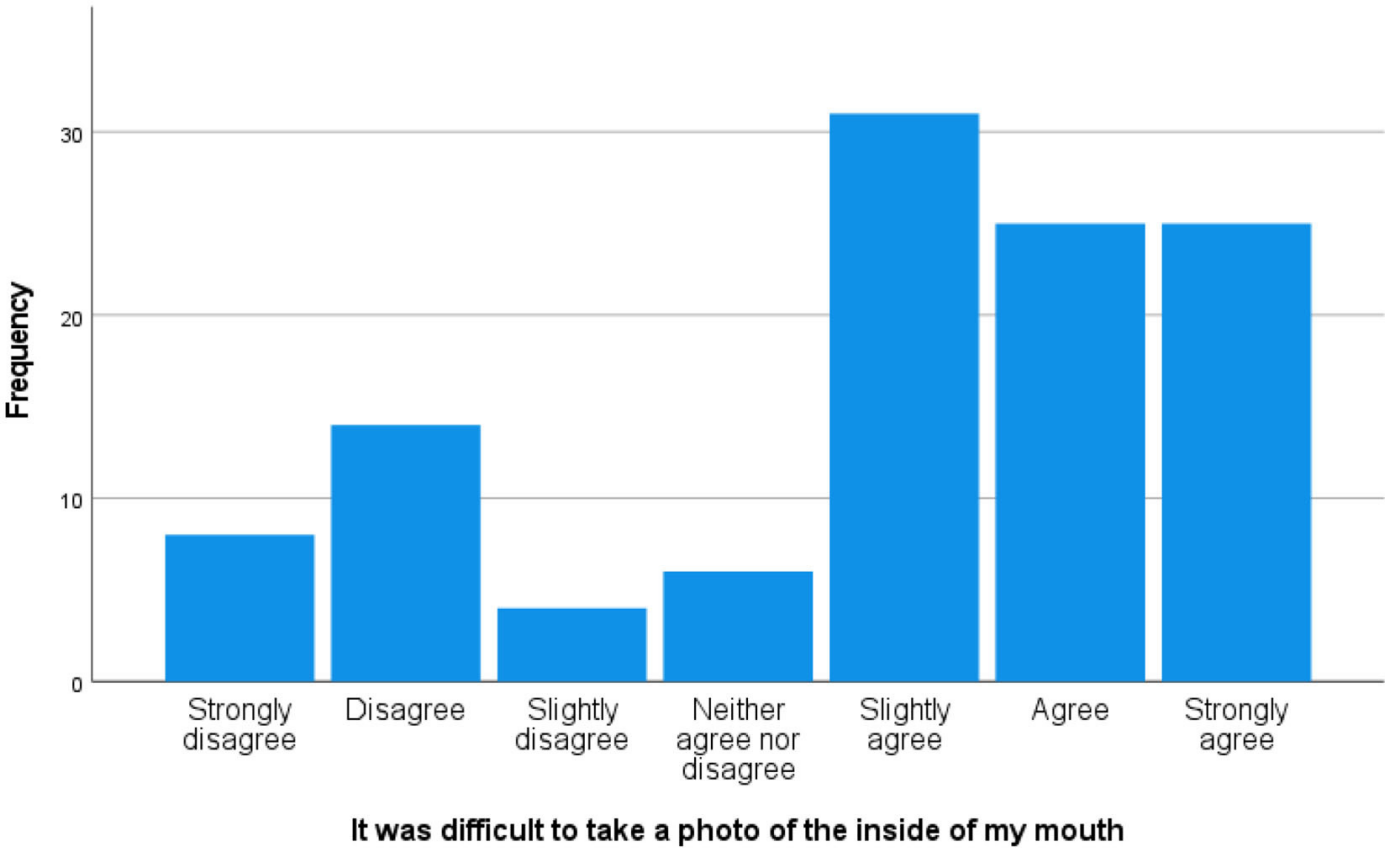
Reported difficulty of taking an “oral selfie.”

The great majority (93.80%) had taken the photos themselves, and a few were taken by their partners or spouses. The mean ± SD number of photos that had to be taken to get 1 uploaded was 8.28 ± 6.55, range 1–34. Time taken was a mean of 6.80 ± 6.41 min, range 1–45. The majority (75.20%) reported using Apple phones, 15.9% Samsung, and the remainder a variety of Android smartphone makes. Figure 2 illustrates the reported difficulty of obtaining the photograph, with a mean, median, and mode of “slightly difficult.”

**Figure 3:**
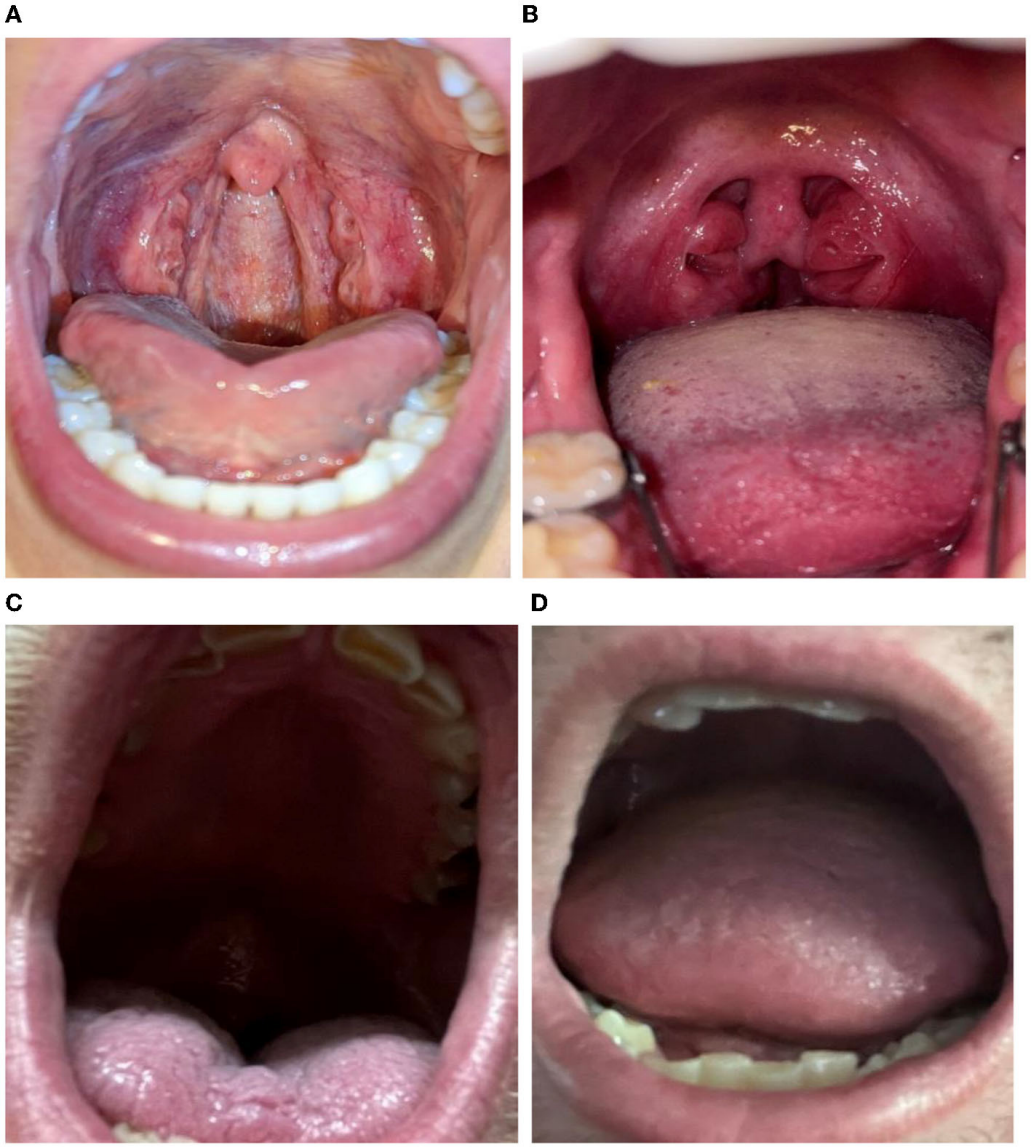
“Oral selfie” examples. **(A)** Good quality. **(B)** Good quality, inflamed tonsils. **(C)** Too dark. **(D)** Occluded oropharynx, poor focus.

Figure 3 illustrates that high-quality oropharyngeal selfies can be obtained from men through limited instruction such as that provided here (Figure 3A). Figure 3B illustrates that one of the participants had bilateral tonsillitis, and that enlarged tonsils are clearly visible. There were also a number of unsuccessful images, for reasons of being too dark (Figures 3C, D), having the oropharynx occluded by the tongue (Figure 3D), and being poorly focused (Figure 3D).

There were significant correlations between the quality ratings of the three reviewers blind to each other's ratings. The otolaryngologist was the “gold standard” reviewer and gamma coefficients with the medical researcher were 0.88, *p* < 0.001, and the dentist, 0.72, *p* < 0.001.

Quality ratings by the otolaryngologist were as follows: good or acceptable quality, 22.1%; oropharynx partially occluded, 29.2%; oropharynx not visible, 18.6%; image too dark, 21.2%; and image too small, 8.8%. For these last two categories, an additional secondary code of partial or complete occlusion was registered for 12.

In the second wave, of the 65 whose images were rated as unacceptable by all the three raters in the first wave, 49 commenced the second wave exercise and 46 (70.8%) completed the exercise. The orolaryngologist's blinded ratings on the same scale were of an additional 7 (15.2%) rated good or acceptable quality. However, 36 (78.3%) remained partially obscured. Combining the participant results for the first and second waves, 28.3% of the 113 were rated as good or acceptable images.

## Discussion

These data indicate that GBM will respond to a request for a smartphone oral selfie with email instructions and that oral selfies of high enough quality to identify potentially malignant oropharyngeal lesions can be obtained. Oral selfies of sufficient quality for potential diagnosis can be obtained from GBM. Younger men who were HPV vaccinated, better informed about HPV-related oropharyngeal cancer, and agreed that the benefits of screening for oropharyngeal cancer were higher than for other early cancer screenings and were more likely to return images. This feasibility study indicates that for 28% of SGM, taking oral “selfies” of a quality to potentially observe the potential malignancies and returning them to a study website is feasible with minimal training. However, some participants could do this efficiently while others took significant time. Research that further explores more detailed approaches to training and guided telemedicine to explore oropharyngeal images is clearly warranted.

A serendipitous data image that shows bilateral tonsillitis (Figure 3B) illustrates the level of detail which may be obtained. Relatively simple modifications (an instruction video, using flash in dark photos or better positioning of the camera to improve focus and image size) can increase the proportion of quality oral selfies to over 28% in total by improving some second-wave previously unacceptable quality images.

It is clear that these quality images may be obtained by oropharyngeal selfies, using only online instructions, or as in the second wave, a video demonstration with instructions. The most difficult image modification is to prevent the tongue from occluding the palatine tonsils, which, along with the tongue base, constitute the majority of potentially malignant lesions. Those which were too dark could be remedied by the use of flash; and too small, by better positioning of the camera. Clearer instructions, using an instructional oral selfie video for those whose oral selfie was judged inadequate by any of the 3 raters of the first wave, resulted in an additional 15.2% of the second wave being judged acceptable by the otolaryngologist. In this second wave, the fault remaining was partial occlusion of the palatine tonsils by the tongue. Clear video instructions do appear to raise the proportion of quality images by 15%, in those who previously had inadequate images. The total number of acceptable images of the first and second waves, by the participant, was 28.3%.

Inter-individual variability in tongue bulk and “size” of the oral cavity will likely mean that some subjects will not benefit from this approach. Furthermore, given that most tongue base tumors are not readily visible via trans-oral view, our approach will not allow screening for this sub-site. However, given that the bulk of HPV-related oropharyngeal tumors occurs in the palatine tonsils and visible oropharynx, oral “selfies” do appear to provide an opportunity to economically screen online, without having to rely on clinic attendance, which may be geographically or economically challenging for GBM, many of whom may not wish to “come out” to a clinician or who may anticipate stigma or discrimination, have heightened concerns about confidentiality, or not want to discuss their sexual behavior or identity.

In potentially malignant HPV-associated oropharyngeal lesions, their significantly improved outcomes over classic oropharyngeal lesions (5, 6) make screening attractive, especially in high-risk populations such as GBM. Progress in using AI to identify oral lesions requiring referral suggests that may be possible to identify suspicious oropharyngeal lesions in GBM.

While HPV vaccination will over time reduce the incidence of HPV-related cancers, it is calculated that the full effect of HPV vaccination will not occur until the year 2045 (1). Interventions to obtain oropharyngeal “selfies” can also be used to encourage HPV vaccination, but HPV vaccinations (a course of 3 over 6 months) are costly in the US, and not recommended for men over the age of 45 years. Potentially, oral “selfies” may open the possibilities of online screening for those who fall through the gaps of receiving HPV vaccination.

While this study was limited to oropharyngeal selfies to identify possible HPV-related malignancies, they do also suggest that *oral* selfies of the oral cavity and buccal mucosa, palate, and gingiva may be possible. Given that GBM report relatively high rates of alcohol and tobacco use, both related to higher rates of oral malignancies (10, 11), it may be possible to screen for *oral* lesions, although this was not explored in the present study.

These pilot data are limited by being a random sample of a larger study in the US. There are inadequate geographic data on the rise in HPV-related oropharyngeal cancer in men, and so these findings may not be generalizable beyond Western countries (18). Furthermore, few major cancer registries ask questions on sexual behavior or sexual identity, severely limiting data available for epidemiological analysis. Nevertheless, it appears that although not all oral selfies in this study are of a quality to make diagnoses, the potential of oral “selfies” as a screening tool for HPV-associated oropharyngeal lesions is worth further exploration.

## Data availability statement

The datasets presented in this article are not readily available because, the study is still ongoing. Requests to access the datasets should be directed to mwross@umn.edu.

## Ethics statement

The studies involving human participants were reviewed and approved by University of Minnesota IRB. The patients/participants provided their written informed consent to participate in this study.

## Author contributions

MR: wrote draft, conducted data analysis, and grantee. SB: curated data and conducted data analysis. CN, SK, and MR: rated clinical images. IZ, JW, BR, CS, AN, CF, CN, SK, and SB: reviewed and edited versions of the drafts. All authors contributed to the article and approved the submitted version.
